# Extraction of Land Information, Future Landscape Changes and Seismic Hazard Assessment: A Case Study of Tabriz, Iran

**DOI:** 10.3390/s20247010

**Published:** 2020-12-08

**Authors:** Ayub Mohammadi, Sadra Karimzadeh, Khalil Valizadeh Kamran, Masashi Matsuoka

**Affiliations:** 1 Department of Remote Sensing and GIS, University of Tabriz, Tabriz 5166616471, Iran; mohammadi.ayub@tabrizu.ac.ir; 2 Institute of Environment, University of Tabriz, Tabriz 5166616471, Iran; 3 Department of Architecture and Building Engineering, Tokyo Institute of Technology, Yokohama 226-8502, Japan; matsuoka.m.ab@m.titech.ac.jp

**Keywords:** remote sensing, GIS, Markov chain, land use, urban information, Tabriz City

## Abstract

Exact land cover inventory data should be extracted for future landscape prediction and seismic hazard assessment. This paper presents a comprehensive study towards the sustainable development of Tabriz City (NW Iran) including land cover change detection, future potential landscape, seismic hazard assessment and municipal performance evaluation. Landsat data using maximum likelihood (ML) and Markov chain algorithms were used to evaluate changes in land cover in the study area. The urbanization pattern taking place in the city was also studied via synthetic aperture radar (SAR) data of Sentinel-1 ground range detected (GRD) and single look complex (SLC). The age of buildings was extracted by using built-up areas of all classified maps. The logistic regression (LR) model was used for creating a seismic hazard assessment map. From the results, it can be concluded that the land cover (especially built-up areas) has seen considerable changes from 1989 to 2020. The overall accuracy (OA) values of the produced maps for the years 1989, 2005, 2011 and 2020 are 96%, 96%, 93% and 94%, respectively. The future potential landscape of the city showed that the land cover prediction by using the Markov chain model provided a promising finding. Four images of 1989, 2005, 2011 and 2020, were employed for built-up areas’ land information trends, from which it was indicated that most of the built-up areas had been constructed before 2011. The seismic hazard assessment map indicated that municipal zones of 1 and 9 were the least susceptible areas to an earthquake; conversely, municipal zones of 4, 6, 7 and 8 were located in the most susceptible regions to an earthquake in the future. More findings showed that municipal zones 1 and 4 demonstrated the best and worst performance among all zones, respectively.

## 1. Introduction

Rapid urbanization, deforestation and increasing population have led to global environmental changes [1]. Because of this, large areas of agricultural land are being converted into urban land and industrial estates, which are prone to land degradation [1,2]. Typically, urbanization influences climate and water quality [3], which can result in changes in local climate. One of the main means by which to understand the relationship between humans and their environment is recording the changes occurring where they live [3,4]. Useful information can be obtained from the pattern and direction of land cover changes, from which better planning for sustainable development is possible. Landsat satellite imageries are widely used medium-scale data for land surface change analysis [2,5]. The low temporal baseline of these data is considered as a weak point that sometimes results in the omission of some dynamic changes [3]. Urban sprawl significantly changes landscapes across urban areas [6], which is usually associated with changes among vegetation, built-up areas and bare lands in the near or remote future. For seismic hazard assessment caused by an earthquake, urban information is urgently needed [7]. Normally, field checks and digital interpretation using the technology of remote sensing (RS) are among the common ways to extract urban information [1,7,8]. Geographic information systems (GIS) together with remote sensing technology can provide useful information for decision-makers [9]. RS data have successfully been applied for mapping and measuring the area and the extent of land cover. Satellite image performance has now been improved to a ground resolution of less than 1 m to be acquired. Physical assessment of urban areas from remotely sensed data enables comparative analysis of a city’s extent within a region [3]. In order to record all changes, selecting the proper temporal baseline of RS data is very important [10]. The Landsat satellite imagery (medium spatial resolution RS data) has widely been utilized to quantitate urbanization across the world [2,11]. 

Predicted land use changes can help land use planners in mitigating the negative impacts on the environment. In recent years, the city and the surrounding areas have been several times shocked by large and small earthquakes. In developed countries, building inventory information is provided by local and central institutes [7]. Nevertheless, in developing countries like Iran, this is quite the opposite, so researchers should work and provide information to different organizations. Normally, this kind of study requires great effort and considerable financial support. There are many faults in Iran, a few of which have recently been activated and claimed many lives and also caused a great deal of damage to properties [12]; because of the possibility of the recurrence of such events in the near or remote future, fear still exists among inhabitants in these areas. Typically, because of the high buildings, this fear is most common among people in large cities like Tabriz.

Efforts have been made to carry out land cover information extraction using RS data and techniques. Urbanization influences ecosystems, but in order to determine and understand these impacts, precise and accurate information about the land cover’s temporal and spatial changes is essential [2,3]. Built-up areas are very important for many studies, including those considering buildings’ age, seismic hazard assessment, and prediction, so as to enable the optimal updating of built-up areas. Maximum likelihood, as one of the best methods for classification [11,13], was employed for extracting land cover using four cloud-free Landsat data. Accordingly, building inventory and urban sprawl information are important factors for damage estimation [7,14,15]. In order to determine if older buildings have been constructed under older seismic hazard standards, a map of the ages of buildings is needed [7,12]. For a fast and effective response during an earthquake, an urgent evaluation is needed [7], but, at present, sufficient information is provided to be used in the future to mitigate possible damages in the study area. Previous research simply studied the land cover detection of the city, but a comprehensive study on land cover change detection, future potential land cover, municipal performance evaluation and seismic hazard assessment is missing for Tabriz, which is one of the largest and most important cities in Iran. Therefore, this study presents a more comprehensive examination of the study area compared to the previous research, making it highly significant.

Many studies conducted land cover change detection and prediction using different models of fuzzy logic modeling [16,17,18,19,20]; geo-statistical methods [21,22,23,24]; Markov-CA [5,10,25,26,27,28,29,30,31,32]; cellular automata models [33,34,35,36]; propagating aleatory and epistemic uncertainty [37,38]; artificial neural network [39,40,41,42,43]; Hopfield neural network [44,45,46,47,48]; supervised back-propagation neural network [49,50]; self-adaptive cellular based deep learning [51,52,53,54]; analytical hierarchy process [27,55,56]; geographic information system (GIS)-based hybrid site condition [15,57,58]; recurrent neural network [59,60,61,62]; change vector analysis [63,64,65,66,67]; and different satellite imageries of both SAR and optical [1,2,3,6,7,9,10,11,13,14,68,69,70,71,72,73,74,75,76].

Previous studies on Tabriz City were not comprehensive, failing to include seismic hazard assessment and municipality performance. In recent years, the rapidly increasing population of the city has necessitated the construction of high-rise buildings and conversion of agricultural land into built-up areas. A comprehensive study that exploits the advantages of both remote sensing and GIS can turn satellite data into an actionable level that can be used for proper environmental planning. For the study area, a comprehensive evaluation from the point of view of satellite datasets and GIS is lacking. To fill this gap, GIS layers of various spatial information, SAR images from Sentinel-1 and optical images from Landsat missions were gathered to perform a new study of land cover change detection, future potential landscape, distribution of buildings by age, municipal performance evaluation and building damage assessment in Tabriz.

## 2. Description of the Study Area

**Figure 1:**
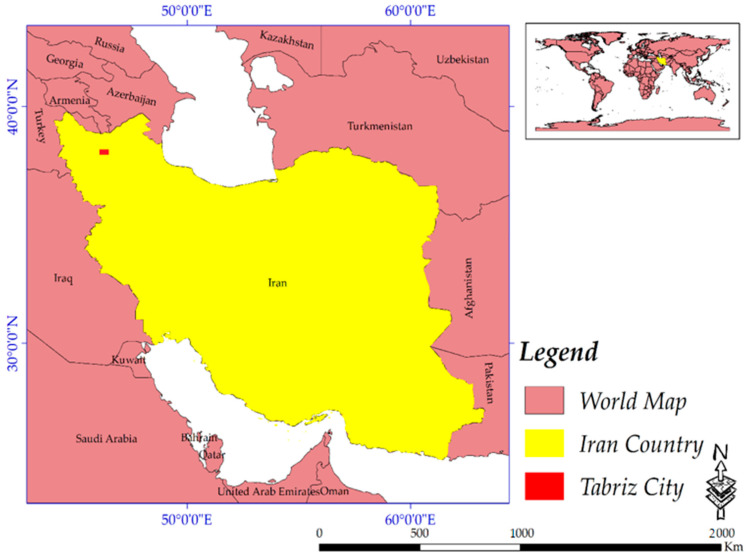
The geographical extent of the city.

The study area (with a population of over 1.7 million and area of 321.03 km^2^) is the metropolitan area of Tabriz, East-Azerbaijan, Iran, defined by its 10 municipal regions (Figure 1). Tabriz City is located in Azarbaijan geological zone, which is surrounded in the northern region by Alborz, in the south by Semnan and in the west by the Tabriz–Urumiyeh Faults [77]; therefore, it is considered as an area susceptible to earthquakes. Tabriz City continues to the Pontic highlands in Turkey [77,78]. The central and western regions of Iran are comparable with the Azarbaijan geological zone [79], where there are a few important faults [15,78,79,80]. The Tabriz fault is the most important one near to the city of Tabriz, extending in the northwest–southeast direction from the Zanjan zone and continuing to the northern mountains of Tabriz City [78]. It has been selected because: (1) it is the 5^th^ large city of Iran which is located near the Tabriz fault, and in recent years, the land cover has rarely been updated; (2) it is a hub for cities in the northwest and west of the country (mainly due to better facilities including more job opportunities, higher quality education and more health centers, more people tend to migrate to the city); and (3) for such cities with this level of importance, future land cover prediction and seismic hazard assessment is vital. Seasons in Tabriz are regular, and it has a continental and cold semi-arid climate; at the same time, the average annual precipitation in the study area is around 320 mm, while the average annual temperature is almost 12.6 °C [81,82]. The city experiences humid and rainy weather in autumn, while it has a few snowy days during the winter season; at the same time, in spring, the city has a mild and fine climate, and during summers, the region can experience a semi-hot climate [81,82].

## 3. Materials and Methods

### 3.1. Database and Data Acquisition

#### 3.1.1. Optical Satellite Data

For the study area, we obtained four cloudless satellite data, from which a Landsat-5 image of the year 1989 was selected as the base image for the study. All the Landsat images were downloaded through the USGS portal. Based on the metadata, only the data with cloud coverage of less than 10% were selected. Then, we searched for and collected RS data between 1989 to 2020, and cloudless Landsat-5 data for 2005 and 2011 were found. Regarding data for the year 2020, an image of Landsat-8 operational land imager (OLI) was collected. The images include seven bands in the range of visible to thermal-infrared for Landsat-5 and nine bands for Landsat-8. The ground resolution of images in optical bands is 30 m, except for a panchromatic band of Landsat-8, which is 15 m. The technical characteristics of the Landsat data are clearly presented in Table 1.

#### 3.1.2. Synthetic Aperture Radar data

The study was focused on the Landsat missions to carry out the objectives, but the SAR data of Sentinel-1 (SLC and GRD) were also employed for extracting land cover, especially built-up areas, because built-up areas are associated more with seismic hazard assessment; therefore, the validity and reliability of its extraction should be taken into account. Table 2 shows the geometric attributes of SAR data.

### 3.2. Data Preprocessing and Processing

**Figure 2:**
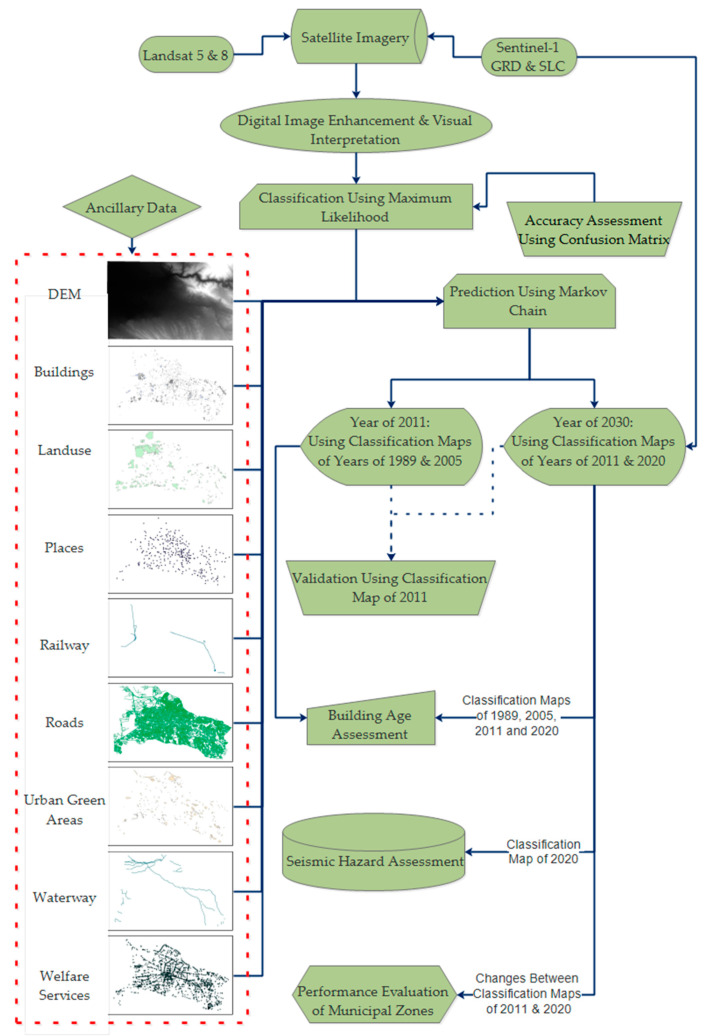
The methodology of the research.

First of all, all data of Landsat-5 and 8 were processed for atmospheric, radiometric corrections and the spatial resolution of them was enhanced to 15 m using a panchromatic band of Landsat-8. It is worth mentioning that in pan-sharpening, spectral information will remain unchanged, while the spatial resolution of higher pixel size images will be assigned to the lower one. Here, the 30-m spatial resolution of the Landsat-5 data was enhanced to just 15-m using the panchromatic band of the Landsat-8. These data were imported into TerrSet Software for classification, change detection and prediction using the Markov chain algorithm for the years 2011 and 2030. At the first stage, the regions of interest (ROIs) were extracted carefully; then, by the maximum likelihood algorithm, changes in land cover from Landsat satellite images were detected, classified and mapped. Furthermore, all ancillary data were processed and applied together with classified maps for the prediction steps using the Markov chain model. The overall approach for the current research includes three key procedures: (1) geometric correction of data; (2) classification of optical satellite data and prediction of the future potential landscape of the city; and (3) municipal performance evaluation and seismic hazard assessment (Figure 2). Ancillary reference data were collected from the Municipality of Tabriz and open street map (OSM) and were applied for training the Markov chain algorithm. These data included a digital elevation model (DEM), buildings, land use, places, railway, roads, green space, waterway and welfare services shapefile of the city (Table 3). Additionally, those data which were collected from the municipality of Tabriz were up to date and were based on the latest changes that occurred in the city.

### 3.3. Model Used in the Study

#### 3.3.1. Maximum Likelihood

A maximum likelihood classifier was applied to extract surface information from RS data. This defined the statistical values with a normal distribution for each class in image’s bands. On the other hand, the algorithm estimated the probability that one pixel would fall into the defined classes. This procedure was continued for all the pixels and the pixels were assigned for those classes that produced the highest probability as follows [83]:

(1) gi(x)−1n p(∞i)−121n|Σi|−12(x−mi)∑i−1 (x−mi)

where the number of classes is defined by $\;g_{i}$ (x), which represents the number of imageries’ bands, p$(\infty_{i}$) describes the probability of class, which occurs in the images, the covariance matrix is defined by $\left|\sum_{i}\right|$; additionally, the inverse matrix is $\underset{i}{\sum }-1$, and $m_{i}$ is the mean vector [83].

#### 3.3.2. Markov Chain

Markov chain is a model from which the future potential landscape can be relatively detected, so that, based on the extracted information from the past data, it detects the future pattern of a land cover [25,26,27]. In this countable sequence, the chain moves state at discrete time steps [84,85]. It is worth mentioning that this sequence of time process is called a sequence-time Markov chain [84,86]. Markov chains have many applications in different fields. Overall, this model for land cover prediction produces promising findings [4,35]. The model calculated the following formulas [25]:

(2) $$p_{ij}=\frac{n_{ij}}{n_{i}}$$

(3) $$\overset{k}{\underset{j=1}{\sum }}p_{ij}=1$$

where transition probability is defined by $p_{ij}$, *i* and *j* describe two types of land cover, the total of pixels of each class is shown by $n_{i}\;\mathrm{and}\;n_{ij}$, which represents the number of transformed pixels from class *i* to class *j*, and finally, *k* defines the number of land cover classes.

#### 3.3.3. Logistic Regression Model

A good deterministic seismic hazard assessment is generally associated with effective and well-approved models [16]. In recent years, the LR model has been widely employed to analyze binary variables and, as a result, it has been introduced as an promising approach in environmental studies [19,42]. Therefore, the LR classification model was adopted in this study. It deals with independent and dependent parameters, where this relationship is nonlinear and can be calculated as Equations (4) and (5) [87,88]:

(4) $$P=\frac{1}{1+e^{-Z}}$$

where P is an earthquake’s probability occurrence (0≤P≤1), and $e^{-Z}$ is a linear logistic factor (−∞≤P≤+∞) that is calculated based on Equation (5) [87]:

(5) $$Z=\log\;it\;\left(p\right)=ln\left(\frac{p}{1-p}\right)=b_{0}+b_{1}x_{1}+\cdots +b_{n\;}x_{n}$$

where *Z* is a linear logistic factor, *p* is an earthquake’s probability occurrence, *n* is the number of conditioning variables, and $b_{0}$ is the constant coefficient.

##### Factors Used for Seismic Hazard Assessment

**Figure 3:**
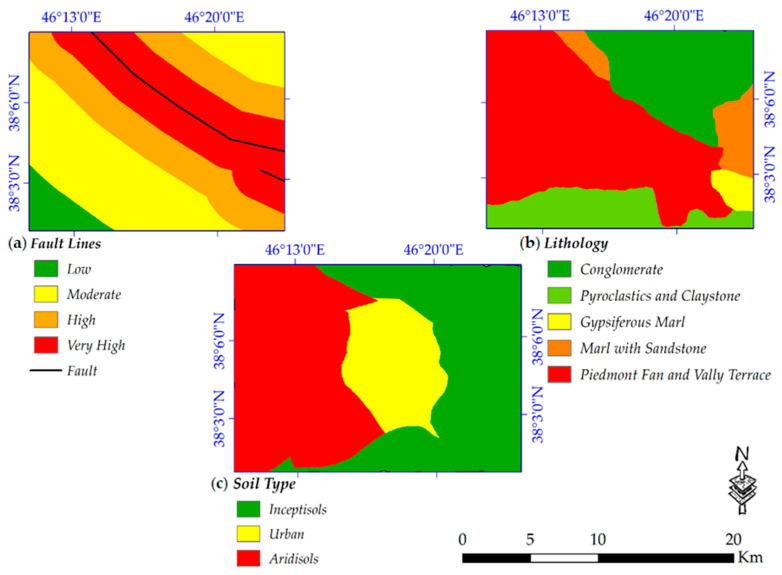
Conditioning parameters used for susceptibility mapping (**a**) fault lines; (**b**) lithology; and (**c**) soil type.

Hazard studies give valuable information about human environments, which, if the results of these studies are taken into account, may protect them from such events in the future. Complex natural hazards such as landslides or flood mapping need considerable data collection and analysis [19,89]. However, in this study, a susceptibility map of potential sites of earthquakes is produced, in which the most important conditioning parameters for it are soil type, proximity to fault lines and lithology condition (Figure 3). A simple probabilistic seismic hazard analysis (PSHA) model was used, which is a useful algorithm, especially when in situ seismic data are not widely available. In the study area, the distribution of the faults is not complex and the Tabriz fault’s orientation is straightforward. However, it must be noted that, due to the lack of actual seismic data, this model cannot address uncertainties well [74]. The occurrence probability and intensity of risk assessment depend on selected conditioning factors [17].

### 3.4. Accuracy Assessment and Validation

#### 3.4.1. Confusion Matrix for the Classified Maps

**Figure 4:**
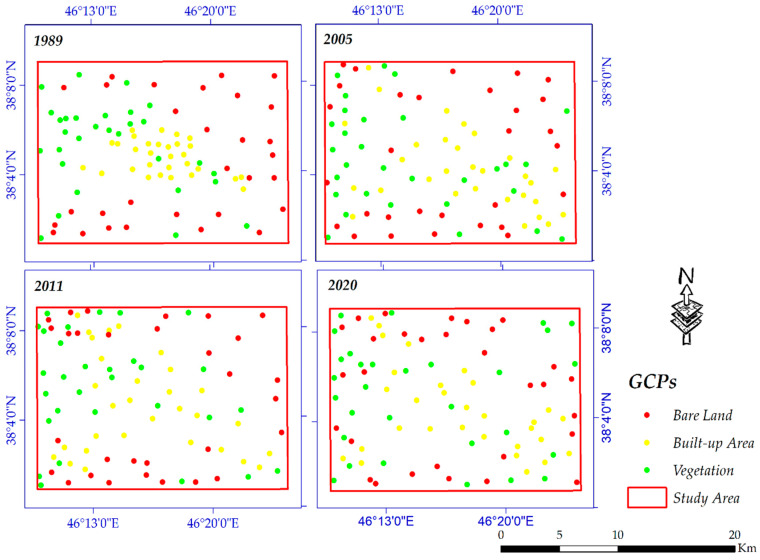
GCPs for all years.

Using Google Earth (GE) images, thirty (altogether ninety) ground control points (GCPs) were randomly extracted for each land cover (Figure 4) and then converted into ROIs for accuracy assessment. The overall accuracy and Kappa coefficient were employed in this research. These two models are measured based on Equations (6) and (7), respectively [90,91]:

(6) $$OA=\frac{1}{N}\sum p_{ii}$$

where *OA* defines the total accuracy of the model, test pixels are described by *N*, and $\sum p_{ii}$ represents the total number of correctly classified pixels.

Kappa coefficient is a statistical model that is employed to measure the reliability of the qualitative items [91]. It is universally accepted that this model is a more robust method than the simple calculations. The Kappa coefficient provides reliable and valuable information for the findings obtained. *OA* must be calculated first in order to measure it.

(7) $$K=\left(OA-\frac{1}{q}\right)\left(1-\frac{1}{q}\right)$$

where *OA* defines the total accuracy of the model; *k* and *q* are Kappa coefficient and unclassified pixels, respectively.

#### 3.4.2. Validation of the Predicted Map of the Year 2011

The predicted land cover map using the Markov chain model for 2011 was validated by the generated land cover map of the same year. This was only conducted to determine the reliability and accuracy rate of the model that will be used for the prediction of the future landscape of the year 2030.

#### 3.4.3. Validation of Extracted Land Cover Using SAR Data

GRD and SLC products of Sentinel-1 SAR data were used for the validation of mapped land cover. For this reason, a pair of SLC products for two close dates was preprocessed in sentinel application platform (SNAP) software and used for the extraction of land cover using RGB creation in the GIS environment. At the same time, the GRD product was also applied for this matter in order to ensure the complete reliability of the mapped land cover; the reliability of the land cover was essential because it was used to create many maps for the study area.

## 4. Results

### 4.1. Land Cover Classification

Reliable detection of landscape change using remote sensing data must strike a balance between affordability and product accuracy [78,92,93]. By applying the maximum likelihood method, vegetation, built-up and bare land surfaces in 1989, 2005, 2011 and 2020 were extracted for the study areas. This information was utilized to create a few maps of buildings by age, municipality performance and seismic hazard assessment. ROI extraction is one the most important steps in land cover classification, from which exact land cover can be extracted, and it also affects the overall accuracy [70,71,94]. Table 4 and Table 5 detail the number of pixels and ROI separation characteristics, respectively. The mean pixel count of the extracted ROIs was used for obtaining the spectral signatures of the land cover. Therefore, an image-derived technique was applied for the extraction of the spectral signatures.

**Figure 5:**
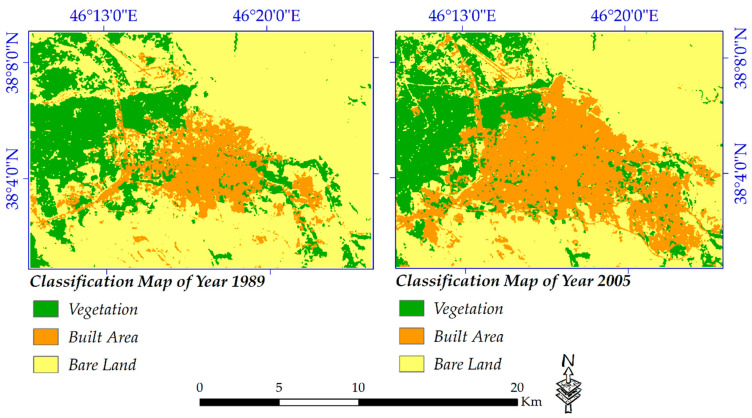
Spatial distribution of changes from 1989 to 2005.

**Figure 6:**
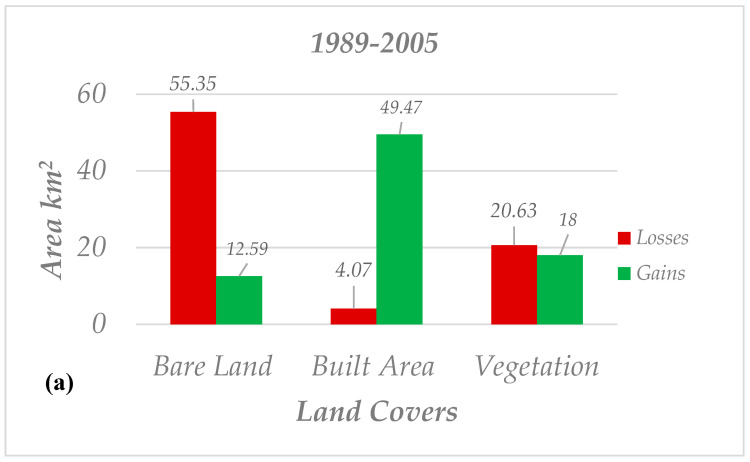

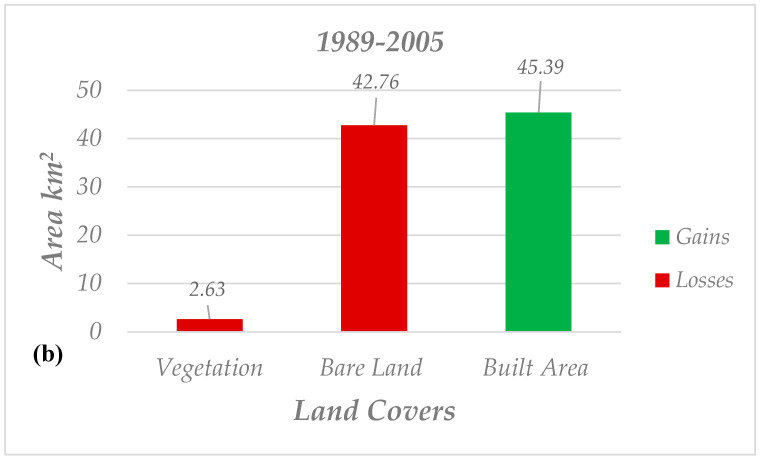
Statistical attributes for changes which occurred from 1989 to 2005; (**a**) gain and loss (**b**) net changes.

The land cover maps for years 1989, 2005, 2011 and 2020 were generated using the maximum likelihood algorithm. Most of the new built-up areas occurred at the edges of the existing urbanized regions, which are displayed in orange color. Figure 5 details the spatial patterns of classified land cover from 1989 to 2005. The vegetation extent on the map is presented in green, built areas in orange and bare land in light yellow pixels. Figure 6 quantifies the changes which occurred from 1989 to 2005. For the sake of distribution clarity, two column charts of gain/losses and net changes were created for changes that occurred from 1989 to 2005. Moreover, vegetation lost around 20 km^2^ and gained 18 km^2^ from 1989 until 2005 (net change −2.63 km^2^). At the same time, 49.47 km^2^ was added to the built-up area, while only 4.07 km^2^ was removed from it (net change +45.39 km^2^). Finally, compared to built-up areas, losses for bare land are considerable, so that it lost around 55.35 km^2^ from its areas and approximately 12 km^2^ was added to bare land (net change −42.76 km^2^).

After visual inspection, general information over the city was gathered; the only drawback of our classification using the maximum likelihood algorithm was considering the airport band of Tabriz City as a built-up area. This bias may be because of the similarity of backscatters for roads inside the built-up areas with the airport band; however, this is a negligible area and can be addressed using simple editing using GIS.

**Figure 7:**
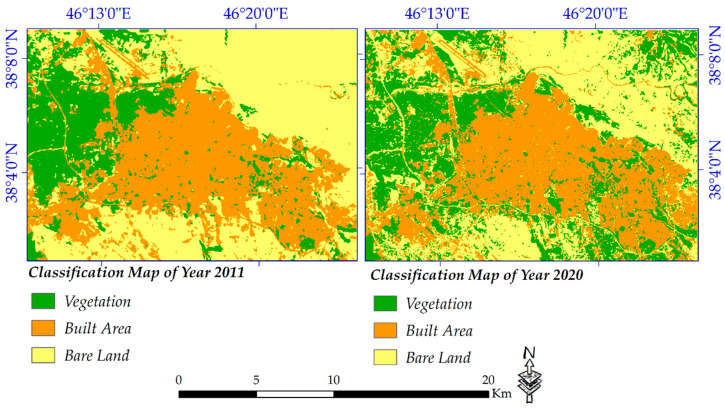
Spatial distribution of changes from 2011 to 2020.

**Figure 8:**
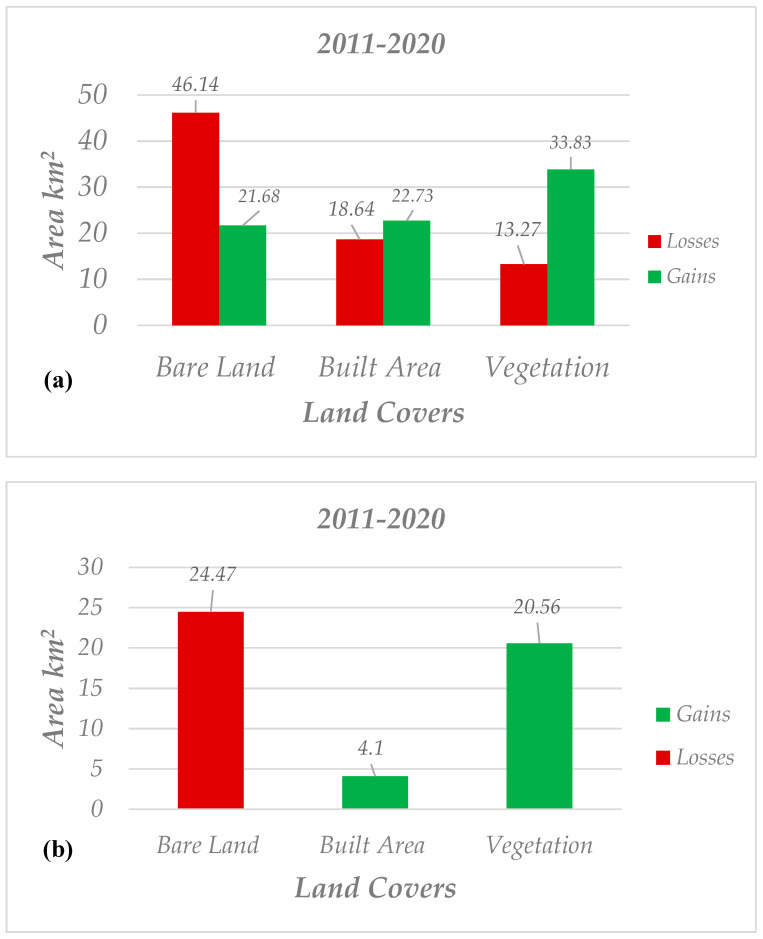
Statistical attributes for changes which occurred from 2011 to 2020; (**a**) gain and loss (**b**) net changes.

Figure 7 represents the spatial trends of land cover from 2011 to 2020, from which increasing vegetation coverage inside the city and beyond is considerable. Two charts of gain/losses and net changes were also provided for changes which occurred from 2011 to 2020 (Figure 8); from these, it can be concluded that from 2011 onwards, only around 4 km^2^ was added to the built-up areas. For many readers, this should be of great concern, but, based on an interview with the municipality of Tabriz (the interview was performed with a public affairs officer of the municipality on 18/08/2020 through phone call), from 2011 onwards, the city’s buildings were constructed and grew vertically, meaning that old buildings with one or two floors were replaced by buildings with more than three floors. A summary of statistical reports for land cover changes from 2011 to 2020 is as follows: (1) net change for vegetation coverage was +20.56 km^2^, meaning that approximately 33.83 km^2^ was added to it and 13.27 km^2^ was subtracted from it; (2) 18.64 km^2^ was subtracted from built-up areas, while 22.73 km^2^ was added to it; and (3) not surprisingly, bare land lost 46.14 km^2^ and gained 21.68 km^2^ so that the net change for it can be −24.47 km^2^.

**Figure 9:**
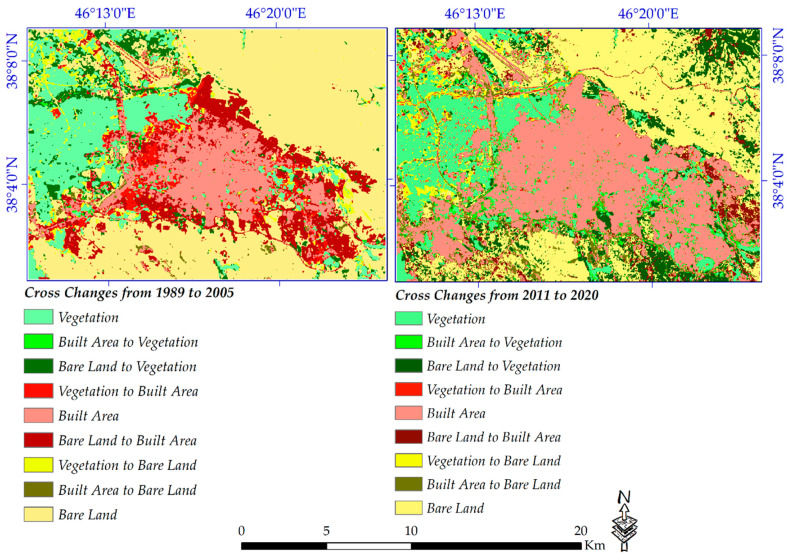
Cross change map of land cover.

Figure 9 clearly shows cross changes from one land cover to the other. For example, those areas that were once built-up areas but were replaced with vegetation coverage are displayed in light green. Dark green represents areas that once were bare land that have become vegetation. The areas indicated by the red color are areas that were originally vegetation but were replaced with built-up areas. At the same time, changes from bare land to built-up areas are indicated by the dark red color. Furthermore, the light yellow color shows areas that were replaced by bare land from vegetation. Additionally, the dark yellow color highlights areas that were built-up areas but then changed to bare grounds. This kind of map is important in showing changes among land cover between two specific years.

### 4.2. Future Potential Landscape of Tabriz Using Markov Chain Model

**Figure 10:**
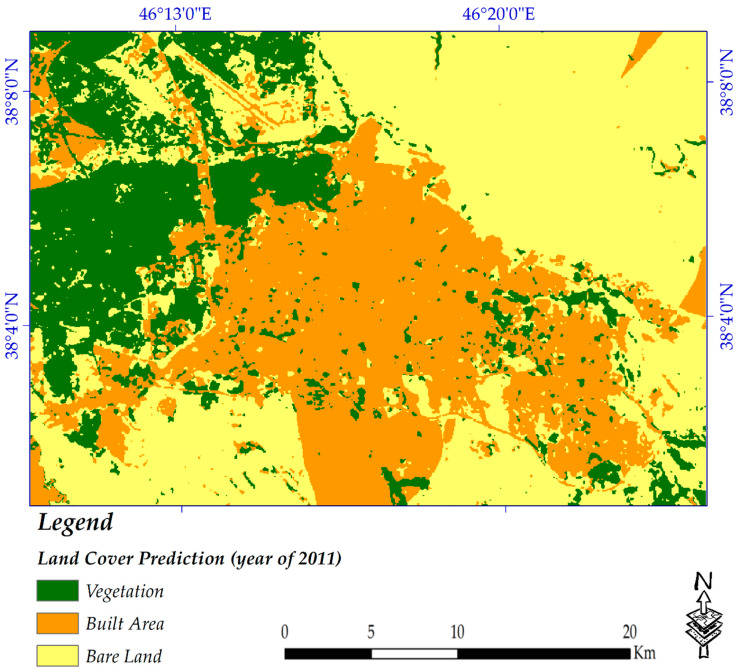
Predicted map of 2011.

The land cover potential pattern of the year 2030 was mapped for the study area. To understand the level of reliability of the model used (because there was no information for the year 2030), the land cover map of the year 2011 was also estimated using land cover maps of the years 1989 and 2005. Considering the predicted land cover map of 2011, around 86% of vegetation coverage was forecasted to remain unchanged (which is a high percentage), and changes from built-up area to vegetation were predicted by approximately 1%, while this rate was roughly 4% from bare land to vegetation. It was forecasted that 96% of the built-up areas would remain unchanged, which is also quite high and shows that the model works well, so the prediction for the year 2030 can be reliable to a great extent. The change prediction rates of vegetation to built-up areas and also the bare lands to built-up areas were estimated to be almost 6% and 8%, respectively. Like vegetation, around 86% of bare land was predicted to remain bare land by the year 2011 (which also represents a good prediction rate). Figure 10 and Table 6 detail the spatial pattern and statistical changes in land cover by the year 2011 (using land cover maps of years 1989 and 2005), respectively.

**Figure 11:**
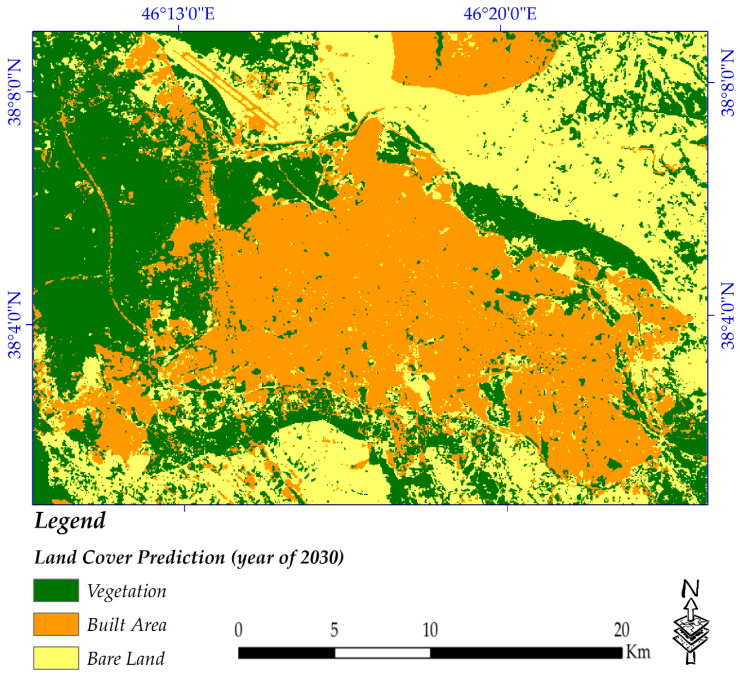
Predicted map of 2030.

Figure 11 and Table 7 highlight the spatial trends and statistical findings of land cover changes by the year 2030, respectively. Considerable findings were extracted regarding the predicted land cover from the map of 2030. Around 74% of vegetation coverage was forecasted to remain unchanged, meaning that almost 26% is likely to be replaced by other types of land cover. Changes from built-up area to vegetation are predicted by approximately 7%, while this rate is quite high for bare land to vegetation, at roughly 26%. It was estimated that 79% of the built-up areas would remain stable as themselves. The change prediction rates of vegetation and bare land to built-up areas are high, at an estimated rate of almost 9% and 11%, respectively. Approximately only 61% of bare land is likely to remain bare land by the year 2030, meaning that based on the changes which occurred until the year of 2020, the municipality plans to change bare land to other types of land cover. One of the considerable results in this regard could be the probability of changes from built-up areas to bare land, at approximately 13% (which is a quite high figure); based on the interview with the municipality, this is maybe because of the reconstruction of buildings over the city that occurred and was recorded by the satellite images used in this study. However, these are only predictions based on changes from the year 2011 to the year 2020.

### 4.3. Building Age Map of the Study Area since 1989

**Figure 12:**
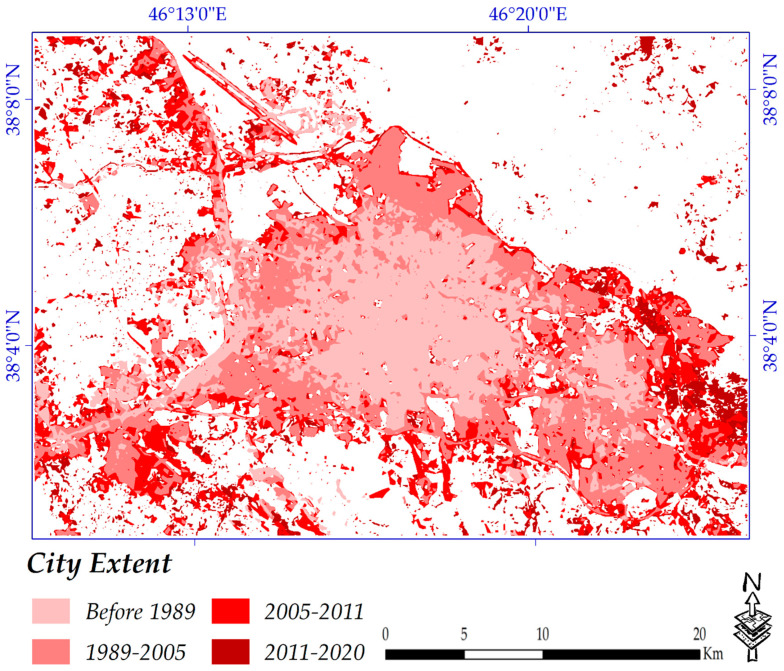
Spatial distribution of building age.

The built-up areas’ distribution by age for the years of 1989, 2005, 2011 and 2020 was extracted (Figure 12 and Table 8). Pixels in light pink color are classified as built-up areas until 1989 and the pink color represents built-up areas that developed after the year of 2005. Areas indicated in red color are defined as newer urban areas that were constructed from 2005 until 2011. The newest built-up areas that have been constructed since 2011 are shown in the dark red color. Most of Tabriz City was constructed before 2011, but relatively new urban areas in and around the study area can be seen, which indicates that urban development has been gradually taking place. The proportion of built-up areas is presented in Table 8, which shows that the total built-up area constructed before 1989 is around 45 km^2^. In the year 2005, the built-up area doubled to approximately 90 km^2^. Almost 21 km^2^ was added to built-up areas by the year 2011. Not surprisingly, only approximately 4 km^2^ has been added to the built-up areas since 2011 (this does not mean that urbanization has stopped since then); this is because buildings have been reconstructed vertically (a few floors) instead of containing only one or two floors. Our interview with the municipality also confirmed that the old buildings with one or two floors are being reconstructed and replaced with buildings with three or more floors. This has good advantages, such as providing more land with the municipality for establishing other projects, while there is sufficient housing for citizens as well.

#### Urbanization Rate

Based on the built-up area extracted from the classified maps in this study, urbanization rate (UR) was calculated using the following user-defined equation:

(8) $$UR=\frac{A}{T}$$

where *UR* is the urbanization rate, *A* is an extended area of built-up areas for each period, and *T* contributes to the time passed for urban growth. According to the equation, *UR* for a different period related to the current study is as follows: it is worth mentioning that the results are km^2^ per year.

$$UR\left(2005\right)=\frac{45.68}{16}=2.85$$

$$UR\left(2011\right)=\frac{21.72}{6}=3.62$$

$$UR\left(2020\right)=\frac{4.11}{9}=0.45$$

### 4.4. Municipal Performance Evaluation for 10 Municipal Zones of Tabriz City

**Figure 13:**
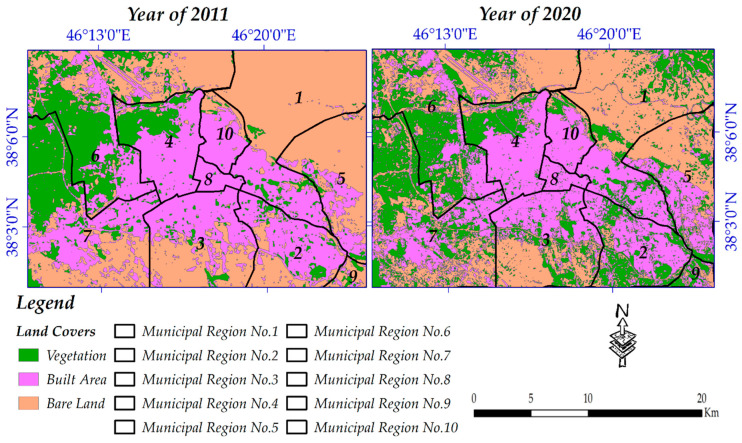
Municipal performance evaluation map.

Based on the changes that occurred from the year 2011 to 2020, the performance of municipal zones of Tabriz city was evaluated (considering the changes towards more built-up areas and green space along with less bare land), meaning that when more bare land for a municipal zone was converted to built-up and vegetation areas, it was considered that the zone worked well. Following our evaluation, the best and worst municipalities are municipal numbers one and four, respectively. Figure 13 and Table 9 show spatial and statistical changes in different types of land cover for each municipality, respectively.

### 4.5. Seismic Hazard Assessment

**Figure 14:**
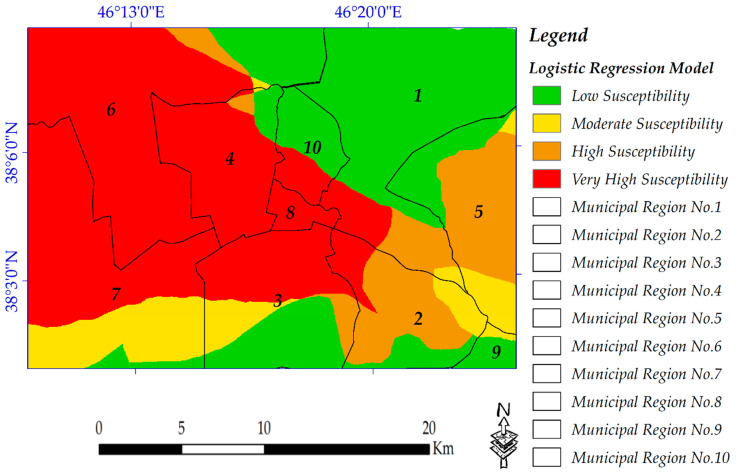
Earthquake susceptibility mapping.

Four susceptible zones were finally reclassified. Figure 14 clearly shows the spatial patterns of areas to susceptible to earthquakes concerning the municipal zones of the city. Most of the municipal zone numbers 1, 9 and 10 are located in the low susceptibility zone, while the entire municipal zone number 8 and most of the municipal zone numbers 3, 4, 6 and 7 are located in the very high susceptibility zone.

### 4.6. Accuracy Assessment and Validation for This Study

#### 4.6.1. Confusion Matrix for the Classified Maps

The land cover classification mapping using RS data is a relatively easy effort, while the accuracy and inaccuracy of it depends on proper data and models used [2,61]. An accuracy assessment using the confusion matrix was accomplished for all four classification maps. The overall accuracy for the classified maps of 1989, 2005, 2011 and 2020 was around 96% 96%, 93% and 94%, respectively. Meanwhile, these values for the Kappa coefficient were almost 93%, 92%, 85% and 88%, respectively (Table 10).

#### 4.6.2. Validation of the Predicted Map of the Year 2011

**Figure 15:**
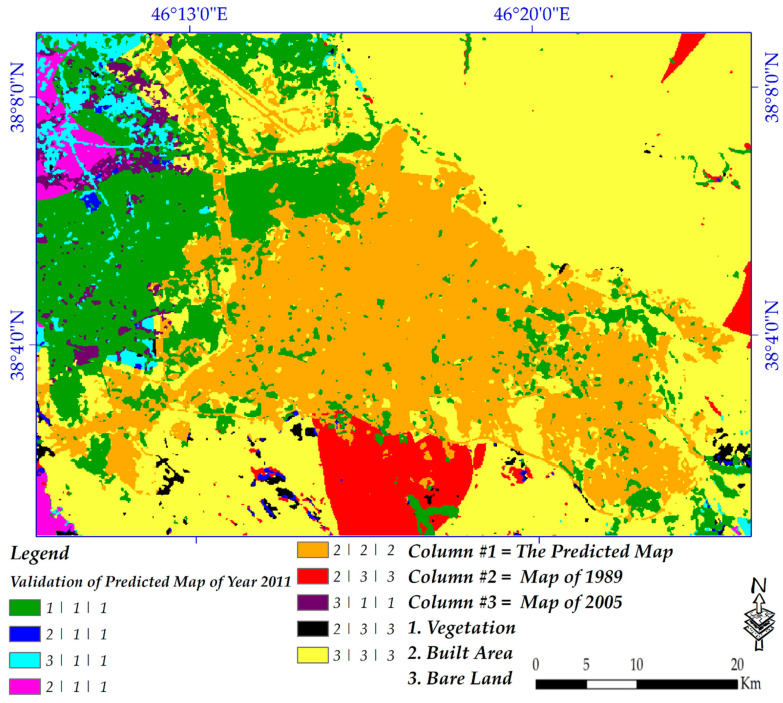
Validation of the predicted map of the year 2011.

Using the land cover map of 2011, the predicted map of the same year was validated. Since there was not any information for the year 2030, the validation was not possible. However, fortunately, the prediction map of the year 2011 was well validated (meaning that it only predicted a few areas incorrectly; most were predicted well); therefore, it can be concluded that the prediction map of the year 2030 can be also correct to a great extent. These land cover products were then used to validate urban extent extraction, which confirmed that land cover extraction was done successfully. Validation interpretation was based on these three data: (1) initial land cover was the classified map of the year 2005 (2); predicted land cover for the year 2011; and (3) validation land cover (classified map of the year 2011). Figure 15 represents the validation of the predicted map of the year 2011. However, for interpretation of the image, two examples are presented here: (1) 1/1/1 means that these areas in all aforementioned images were vegetation, and (2) 2/3/3 means that these areas were bare land originally but were predicted as built-up areas.

#### 4.6.3. Validation of the Extracted Land Cover for the Year 2020 as a Basis for Seismic Hazard Assessment

**Figure 16:**
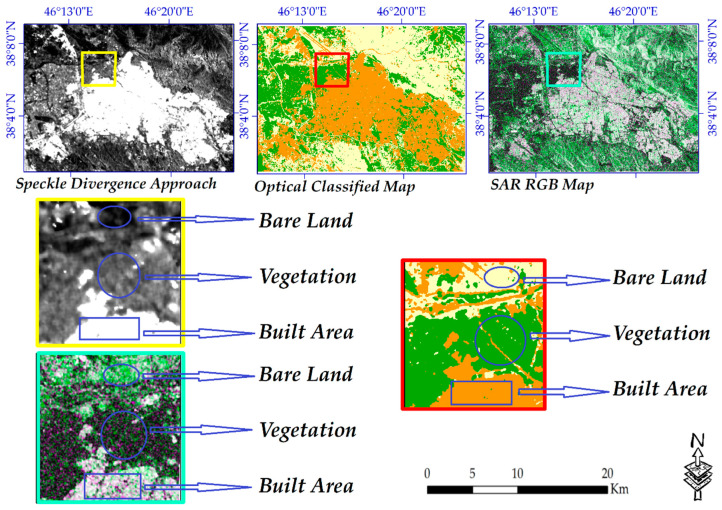
Land cover maps using both optical and SAR data.

The magnitude of errors using conventional methods is a complex issue from which the extracted land cover from them cannot be directly applied for understanding the changes which occurred [3,52]. Additionally, uncertainties are inherent aspects of remotely sensed studies [3]; to minimize uncertainties, SAR data were also applied. This attempt ensured that urban land cover was not missed using optical images. To successfully validate the extracted built-up areas which were utilized for seismic hazard assessment of the city, SAR data were also used. A few small areas were marked by a few geometric symbols (square-shaped) in each set of satellite data (Figure 16). These areas were then enlarged and displayed with different shapes for each type of land cover, comparing the built-up surface from two SAR data of GRD and SLC products, ensuring that the built-up areas in both were successfully matched. After preprocessing and processing of SAR data in the SNAP environment, both bands of VV and VH were employed for RGB creation in the GIS environment. The VV band of the slave imagery was used for R and the VH band of the master one was employed for G and B windows.

## 5. Discussion

Generally, a few diverse factors may affect the results of change detection and prediction [9,84]. The land cover’s spatiotemporal pattern and characteristics in this study were completely different from those which have been measured before, in which a comprehensive study including land cover change detection, future potential landscape, distribution of buildings by age, municipal performance evaluation and building damage assessment was carried out for the metropolitan area of Tabriz, especially for the municipality of Tabriz that has suffered from a lack of such studies. Previous studies were mainly focused on simple methods for measuring the land cover, seismic hazard and building vulnerability for the study area [80,95,96]. During the first period (1989–2005), the built-up area increased as a result of population growth and migration to the city, which was mainly based on destroying vegetation and bare land. During the second period (2011–2020), bare land was replaced by other types of land cover and, in this period of time, one of the most considerable findings was increased vegetation across the city, which, based on the findings, reflects the efforts of the municipal regions to increase the vegetation to a satisfactory level. Urban growth was mainly observed in the bare land and the vegetated areas, far from the economical areas.

The dynamics of the land cover are correlated [77]. Here, built-up areas grew considerably in the first period (1989 to 2005), while in the second period (2011 to 2020), this growth was negligible. For the second period, we found that vegetation areas experienced more positive changes than the first period among land cover. The built-up areas in both periods (1989 to 2005 and 2011 to 2020) showed the largest degree of change among all land covers, which could be linked to the rapid urbanization in Tabriz. The change in speed of bare land was relatively fast in both periods, in which it underwent the most significant land cover changes for the entire period. In addition, vegetation was directly linked to the civil projects of the municipality that turned bare land and old built-up areas into green lands. This analysis suggests that the land cover in Tabriz has considerably changed during the last three decades.

According to the findings for the year 2030, the general trend of change is toward more vegetation and built-up areas as well as less bare land, meaning that the municipality plans to convert more bare land to other types of land cover. Specifically, in the year of 2030, the vegetation and built-up areas will preserve most of their areas and will be larger because of the change from bare land to these types of land cover. Besides this, almost 60 percent of the area of bare land will be preserved, because always bare land will be used for the development of new projects. This implies that the landscape pattern of Tabriz has a tendency to be more optimized in this period.

Based on the results of the urbanization rate, from the year 1989 until 2005, the city of Tabriz experienced growth in urban areas of around 2.85 km^2^ per year. At the same time, this rate was 3.62 km^2^ per year from 2005 until 2011. Additionally, from 2011 to the year 2020, this rate was only 0.45 km^2^ annually.

Seismic hazard assessment is always an essential part of sustainable development projects for urban areas [7,15]. Seismic hazard assessment produces a greater cost efficiency when focusing only on urban areas (rather than the entire area including vegetation and bare land), which has the greatest impact on people when they are destroyed. The application of seismic hazard assessment has not yet been conducted for a city like Tabriz, which is growing fast based on the population rate and its situation as a hub to the other cities of the region. Therefore, a seismic hazard assessment (even a simple one) could enable the local authorities and the policy-makers to direct urbanization to those areas with low or moderate susceptibility. Concerning the earthquake risk for the city, policy-makers should take this into account for future urban sprawl, meaning that they can design stricter policies for new buildings that are constructed or reconstructed in more susceptible areas.

Model validation is important to assess the level of the models’ reliability and validity. Validation of the created maps was performed at three stages. In the first, the classified outputs of the years 1989, 2005, 2011 and 2020 were validated using a confusion matrix. In the second step, and since the land cover map of the year 2020 was selected for damage assessment, two other maps from Sentinel-1 SAR data were also employed. Finally, a classified map of the year 2011 was used to validate the prediction map of the year 2011 (only to assess whether the model could predict the future potential landscape or not, so that its prediction for the year 2020 could also be considered correct).

Based on the findings of this study, for arid and semi-arid regions (like the current study area), the maintenance of the existing vegetated areas rather than planting more grasses and trees in less suitable areas is recommended. Although this study, as well as many previous works, has demonstrated that the remotely sensed data and techniques can be well applied for monitoring changes in cities, we recommend that more high-resolution satellite imageries be used to gain further insights into such changes. Future research should focus on the deep learning techniques for change detection and the prediction of land cover; more details on seismic and risk assessments can also be obtained using deep learning algorithms for the study area. The only major limitation of this study was encountered when obtaining ancillary data from the municipality of Tabriz.

## 6. Conclusions

Cities need comprehensive and innovative plans in order to ensure progress based on sustainable development. Although it is very difficult to obtain absolute results from remotely sensed data, relative findings can be captured, which can be effective for any future planning. This study has emphasized changes in land cover and the future landscape in Tabriz City. Other important issues that the current research was focused on include information on building age, municipal performance evaluation and building damage assessment, which contributes to earthquake damage estimation. This study has also compared the results of optical satellite imagery with SAR data to extract the spatial distribution of buildings for the year 2020, which was the base map to evaluate municipal zone performances and seismic hazard assessment. The main findings of the current study are as follows: Landsat images for the years 1989, 2005, 2011 and 2020 were used to quantify the land cover changes from 1989 until 2020 and the results using the confusion matrix were promising. At the same time, by using and comparing SAR data, the accuracy of built-up areas for the year 2020 was well validated and verified. Referring to the assessment of the distribution of built-up areas by age for Tabriz City, we found that most of the built-up areas had been developed before 2011, and from then onwards, the city has been progressing vertically. Seismic hazard assessment for the future of the city was conducted by using a logistic regression model, from which results indicated that municipal zones 1 and 9 are located inside low susceptibility areas, while municipal zones 4, 6, 7, 8, and also most of zones 3 and 10, are located in highly susceptible regions. Further findings revealed that land cover prediction by using the Markov chain model provided a good opportunity to identify the future potential landscape of the city. Finally, based on the land cover maps of 2011 and 2020, the performances of the municipal zones were evaluated, from which results showed that municipal zone 1 followed by zone 5 have the best performances among all. Besides this, the performance of municipal zone 4 is negligible, as is much of municipal zone 6.

## Figures and Tables

**Table 1:** Characteristics of the optical data used.

Satellite Name	Sensor Mode	Resolution (m)	Path	Row	Date
**Landsat-5**	(Thematic Mapper) TM	30	168	34	30 June 1989
**Landsat-5**	TM	30	168	34	20 July 2005
**Landsat-5**	TM	30	168	34	5 July 2011
**Landsat-8**	OLI	30	168	34	11 June 2020

**Table 2:** Geometric attributes of SAR data used.

Satellite Name	Platform	Product Type	Sensor Mode	Date
**Sentinel-1**	S1A	SLC	IW	11 June 2020
**Sentinel-1**	S1A	SLC	IW	23 June 2020
**Sentinel-1**	S1A	GRD	IW	11 June 2020

**Table 3:** Information on ancillary reference data.

Ancillary Data	Description
**DEM**	30 m SRTM DEM downloaded from USGS website
**Buildings**	Polygon shapefile of buildings collected from the municipality of Tabriz
**Land Use**	Information on recently modified land use of a few place across the city, collected from the municipality of Tabriz
**Places**	Towns, crossroads and squares
**Railway**	Railway shapefile obtained from OSM
**Roads**	Roads shapefile obtained from OSM
**Green Space**	Information of recent made green areas over the city, collected from the municipality of Tabriz
**Waterway**	Waterway shapefile acquired from OSM
**Welfare Services**	Masques, hotels, educational institutes, public parks, sports centers and gyms, banks, petrol stations, hospitals, drug stores, markets and recreational facilities

**Table 4:** Number of pixels used for ROIs.

ROI Summary	Pixel Count: 1989	Pixel Count: 2005	Pixel Count: 2011	Pixel Count: 2020
**Vegetation**	10,311	11,434	10,120	9312
**Built Area**	18,280	17,356	15,670	21,098
**Bare Land**	23,649	25,780	22,456	27,809

**Table 5:** ROI pair separation.

Years	1989	2005	2011	2020
**Vegetation and Built Area**	1.99575610	1.99296923	1.99971843	1.99938326
**Vegetation and Bare Land**	1.99787821	1.99999926	1.99999977	1.99999849
**Built Area and Bare Land**	1.99888996	1.99999990	1.99899203	1.99832448

**Table 6:** Probability of land cover changes in 2011 predicted from maps of the years 1989 and 2005.

	Vegetation %	Built Area %	Bare Land %
**Vegetation**	0.8616	0.0618	0.0767
**Built Area**	0.0138	0.9604	0.0258
**Bare Land**	0.0440	0.0872	0.8688

**Table 7:** Probability of land cover changes in 2030 predicted from maps of years 2011 and 2020.

	Vegetation %	Built Area %	Bare Land %
**Vegetation**	0.7495	0.0915	0.1590
**Built Area**	0.0769	0.7927	0.1304
**Bare Land**	0.2637	0.1198	0.6164

**Table 8:** Area of constructed regions by four different years.

Year	1989	2005	2011	2020
**Area of built-up regions (km^2^)**	45.30	90.98	112.70	116.81

**Table 9:** Quantitative results for municipal performance evaluation since 2011.

No. of Municipal Region	Land Cover	The Year 2011 (km^2^)	The Year 2020 (km^2^)	Percent of Changes	Rate of Change (km^2^)	Performance Rank
1	Vegetation	2.37	11.76	396.2	9.39	1
	Built Area	12.26	13.59	10.8	1.33	
	Bare Land	43.25	32.55	−24.7	−10.7	
2	Vegetation	2.41	8.12	236.9	5.70	3
	Built Area	13.55	14.31	5.6	0.7	
	Bare Land	9.28	2.81	−69.7	−6.46	
3	Vegetation	1.70	6.64	290.5	4.9	4
	Built Area	16.17	14.90	−7.8	−1.2	
	Bare Land	17.80	14.15	−20.5	−3.6	
4	Vegetation	7.28	6.58	−9.6	−0.7	10
	Built Area	17.25	16.82	−2.4	−0.4	
	Bare Land	0.83	1.96	136.1	1.13	
5	Vegetation	1.21	4.72	290	3.51	2
	Built Area	7.51	10.42	38.7	2.91	
	Bare Land	22.88	16.49	−27.9	−6.39	
6	Vegetation	25.07	23.67	−5.5	−1.3	9
	Built Area	14.82	15.74	6.2	0.9	
	Bare Land	26.47	26.94	1.7	0.4	
7	Vegetation	19.18	21.70	13.1	2.5	7
	Built Area	19.38	17.38	−10.3	−2	
	Bare Land	17.81	17.30	−2.8	−0.5	
8	Vegetation	0.03	0.07	133.3	0.04	8
	Built Area	3.85	3.76	−2.3	−0.09	
	Bare Land	----	0.05	----	----	
9	Vegetation	0.59	1.52	157.6	0.93	5
	Built Area	0.78	1.15	47.4	0.36	
	Bare Land	2.31	1.02	−55.8	−1.29	
10	Vegetation	0.38	1.31	244.7	0.93	6
	Built Area	8.18	8.19	0.1	0.009	
	Bare Land	2.31	1.37	−40.6	−0.94	

**Table 10:** Statistical results using the confusion matrix for all classified maps.

Statistical Parameters Year of Classified Maps	Overall Accuracy (%)	Kappa Coefficient (%)
**1989**	96.1354	0.9363
**2005**	96.0151	0.9222
**2011**	93.6413	0.8556
**2020**	94.0700	0.8873
